# Differential distribution patterns and assembly processes of soil microbial communities under contrasting vegetation types at distinctive altitudes in the Changbai Mountain

**DOI:** 10.3389/fmicb.2023.1152818

**Published:** 2023-06-02

**Authors:** Yujuan Kang, Haitao Wu, Yifan Zhang, Qiong Wu, Qiang Guan, Kangle Lu, Yiling Lin

**Affiliations:** ^1^Key Laboratory of Wetland Ecology and Environment, Northeast Institute of Geography and Agroecology, Chinese Academy of Sciences, Changchun, China; ^2^College of Resources and Environment, University of Chinese Academy of Sciences, Beijing, China; ^3^College of Tourism and Geography Sciences, Jilin Normal University, Siping, China

**Keywords:** altitudinal gradient, spatial scaling, assembly processes, soil depth, soil microbiota

## Abstract

Diversity patterns and community assembly of soil microorganisms are essential for understanding soil biodiversity and ecosystem processes. Investigating the impacts of environmental factors on microbial community assembly is crucial for comprehending the functions of microbial biodiversity and ecosystem processes. However, these issues remain insufficiently investigated in related studies despite their fundamental significance. The present study aimed to assess the diversity and assembly of soil bacterial and fungal communities to altitude and soil depth variations in mountain ecosystems by using 16S and ITS rRNA gene sequence analyses. In addition, the major roles of environmental factors in determining soil microbial communities and assembly processes were further investigated. The results showed a U-shaped pattern of the soil bacterial diversity at 0–10  cm soil depth along altitudes, reaching a minimum value at 1800 m, while the fungal diversity exhibited a monotonically decreasing trend with increasing altitude. At 10–20  cm soil depth, the soil bacterial diversity showed no apparent changes along altitudinal gradients, while the fungal Chao1 and phylogenetic diversity (PD) indices exhibited hump-shaped patterns with increasing altitude, reaching a maximum value at 1200 m. Soil bacterial and fungal communities were distinctively distributed with altitude at the same depth of soil, and the spatial turnover rates in fungi was greater than in bacteria. Mantel tests suggested soil physiochemical and climate variables significantly correlated with the β diversity of microbial community at two soil depths, suggesting both soil and climate heterogeneity contributed to the variation of bacterial and fungal community. Correspondingly, a novel phylogenetic null model analysis demonstrated that the community assembly of soil bacterial and fungal communities were dominated by deterministic and stochastic processes, respectively. The assembly processes of bacterial community were significantly related to the soil DOC and C:N ratio, while the fungal community assembly processes were significantly related to the soil C:N ratio. Our results provide a new perspective to assess the responses of soil microbial communities to variations with altitude and soil depth.

## 1. Introduction

The spatial distribution patterns of biome structure and diversity, as well as their underlying mechanisms that regulate spatiotemporal changes are the core theme of biogeography (Green and Bohannan, 2006; Chu et al., 2020). Numerous biogeographical studies have focused on the responses of ecosystems to large-scale environmental changes (Fukami and Wardle, 2005; Walker et al., 2010). Altitude gradients provided a verification platform for models to accurately predict biodiversity responses to global change (McCain and Colwell, 2011). Soil microorganisms play an essential role in biogeochemical cycles within terrestrial ecosystems and are sensitive to environmental changes. Therefore, it is crucial to reveal the fundamental mechanisms underlying the microbial community diversity and distribution patterns to assess their relationships with community stability and ecosystem functions, which are the main central goal in community ecology (Hanson et al., 2012; Meyer et al., 2018). In this context, spatial variations in the soil microbial community at large spatial scales have been continually investigated in different ecosystems, including farmland (Constancias et al., 2015), woodland (Singh et al., 2014; Schnecker et al., 2015), grassland (Baker et al., 2009), tundra (Bjork et al., 2008; Kim et al., 2014), and deserts (Andrew et al., 2012). Studies have highlighted the inconsistent conclusions on the altitudinal distribution patterns of microbial communities, such as monotonous (Bryant et al., 2008; Wang et al., 2011), unimodal (Singh et al., 2012), concave (Singh et al., 2014), and no altitude pattern (Shen et al., 2013). Although the influence of spatial distance on microbial distribution was confirmed (Whitaker et al., 2003; Papke and Ward, 2004), few studies have investigated the impacts of spatial distance on microbial evolution in mountain ecosystems considering the strong diffusion ability of soil microorganisms.

Natural selection, dispersal, drift, species formation, and extinction rates showed the strongest effects on microbial altitude diversity patterns (Kassen and Rainey, 2004; Hanson et al., 2012; Nemergut et al., 2013). Previous studies have showed that microbial communities are influenced by both biotic factors (e.g., terrestrial vegetation characteristics) and abiotic factors (e.g., climatic factors and soil physicochemical properties) (Bezemer et al., 2010; Orwin et al., 2010; Thoms et al., 2010; de Vries et al., 2012). Previous studies have revealed clearly spatial distribution patterns of soil microbial communities and diversity (Green et al., 2004; Fierer and Jackson, 2006; Fierer et al., 2009; Lauber et al., 2009; Nemergut et al., 2011). Soil pH, C:N ratio, and vegetation types were the key factors controlling the altitudinal distribution pattern of soil microbial diversity (Rousk et al., 2010; Shen et al., 2016; Bahram et al., 2018).

The Changbai Mountain area is one of the most biodiversity-rich temperate regions, making it a suitable area for studying biodiversity patterns (Yang and Xu, 2003; Shen et al., 2014; Zou et al., 2017). The main objective of this study is to investigate variations in soil microbial (bacterial and fungal) community structures and their assemblies at four altitudes (800, 1200, 1800, and 2300 m) and two soil depths (0–10 cm and 10–20 cm), as well as their responses to environmental factors on Changbai Mountain. The distance-decay relationships (DDRs) were used to explore changes in spatial turnover rates, while a null model was applied to measure microbial assemblies in different soil samples. The following hypotheses were examined in this study: (i) Altitudinal gradients and soil depths have a substantial impact on the diversities and communities of soil microorganism, as well as the spatial turnover rates of their communities. (ii) Altitudinal gradients and soil depths can distinctly influence the mechanisms of microbial community assembly, which are shaped by distinct environmental factors.

## 2. Materials and methods

### 2.1. Site selection and soil sampling

**Figure 1:**
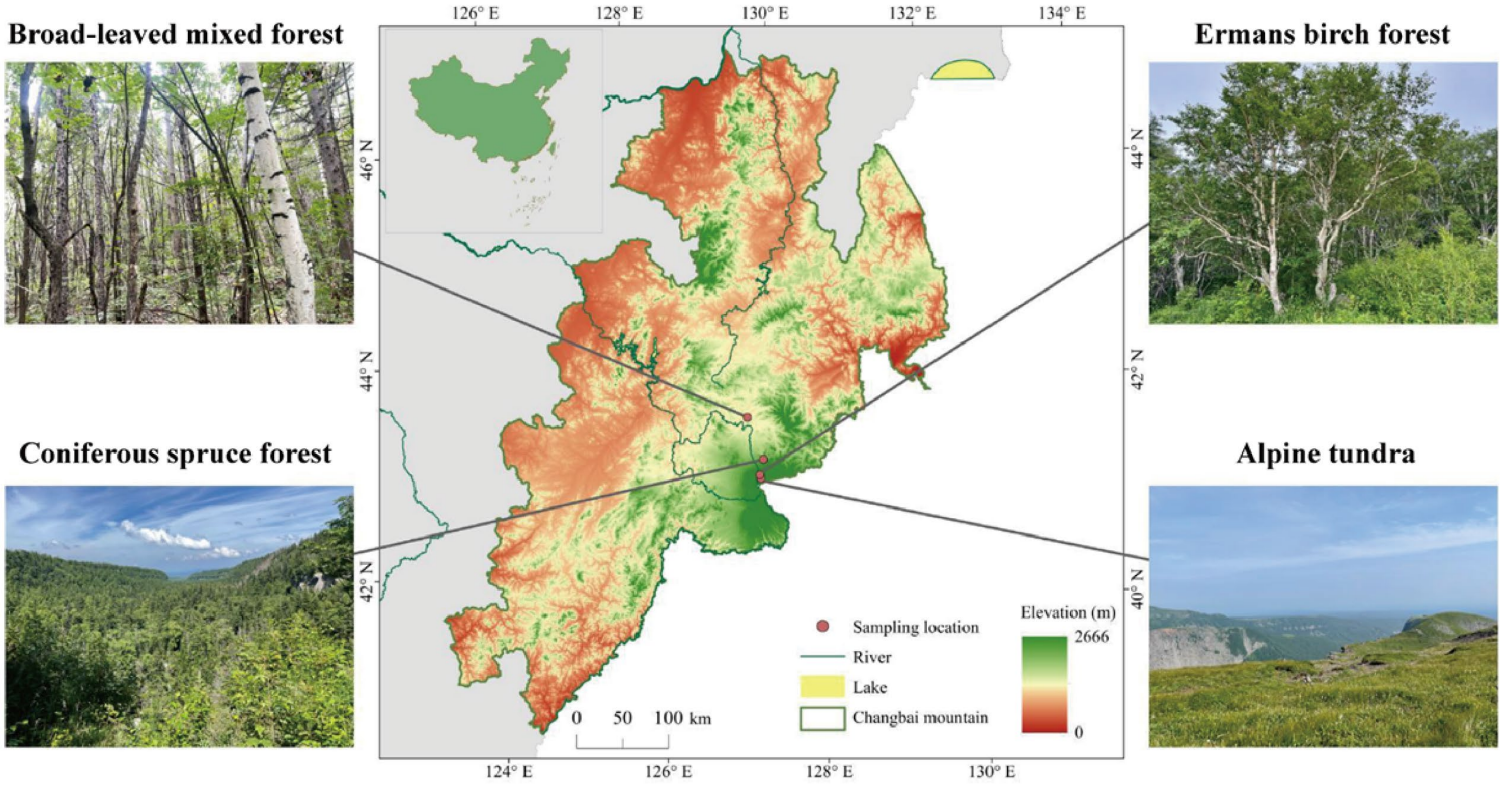
Vegetation types at the study sites on the northern slope of Changbai Mountain, northeast China.

This study was conducted in autumn 2021 on Changbai Mountain (126°55′-129°00′E; 41°23′- 42°36′N), Jilin, northeast China. The study area has a temperate continental monsoon climate with altitudinal variability, whereas the winter is long and cold, and the summer is short and cool. The annual temperature and precipitation range from 2.9 to 4.8°C and 632 to 1154 mm, respectively. The main vegetation types, which exhibit an altitudinal distribution from low 800 m to high 2300 m, consist of mixed coniferous broad-leaved forest (MCB), coniferous spruce forest (CS), Erman’s birch forest (EB), and alpine tundra (AT; Figure 1). The climate and main vegetation characteristics of each habitat are reported in Supplementary Table S1. Three plots (25 m × 25 m each) were established for each vegetation type. To minimize the heterogeneity, we divided each plot into three subplots for soil sampling. The collected soil samples from the three subplots were mixed to obtain one composite soil sample from each plot. In total, 72 subplots were established in the field experiment (4 altitudes × 3 subplots × 3 replicates × 2 depths), from which 24 composite soil samples were collected (4 altitudes × 2 depths × 3 replicates). The collected composite soil samples were placed in plastic bags on ice and transported to the laboratory for further analysis. Soil samples were sieved through 2-mm sieve after the removal of visible debris, such as roots and litter and stored at 4°C for chemical analysis or at −80°C for DNA extraction.

### 2.2. Determination of soil physicochemical properties

Soil pH was measured using a PHS-3C pH meter (PHS-3C pH acidometer) after shaking the soil solution (soil: water = 1: 5) for 30 min. Soil TC and TN contents were determined using a Carlo Erba FLASHEA 1112 CHN-S analyzer according to the instruction manual. Soil total organic (TOC) contents were determined using the external heating-potassium dichromate titration method. Soil total phosphorus (TP) contents were determined using the nitric acid-perchloric acid digestion method combined with a continuous flow analytical system (San++ System, Skalar, Holland) according to the instruction manual. Soil dissolved organic carbon (DOC) contents were determined using a total organic carbon analyzer (TOC VCPH, Shimadzu) according to the instruction manual.

### 2.3. DNA extraction and bar-coded pyrosequencing of the 16S rRNA genes

DNA was first extracted from 0.5 g soil samples using the FastDNA SPIN Kit for soil (MP Biomedicals, Santa Ana, CA, United States), then purified using the UltraClean Soil DNA Kit (MOBIO Laboratories, Carlsbad, CA, United States) following the manufacturer’s instructions. Bacterial 16S rRNA gen V3–V4 region was amplified using the 338F and 806R primers, while the ITS1 region of fungal ITS gene were amplified using the ITS1F and ITS2R primers (Adams et al., 2013). The PCR reactions were performed in triplicate. The PCR product was first extracted from 2% agarose gel, then purified using the AxyPrep DNA Gel Extraction Kit (Axygen Biosciences, Union City, CA, United States) and quantified using an Quantus^™^ Fluorometer (Promega, United States). NEXTFLEX^®^ Rapid DNA-Seq Kit was used to build the library. Purified amplicons were pooled in equimolar amounts and paired-end sequencing was performed on an Illumina MiSeq platform (Illumina, San Diego, United States) in the Majorbio Bio-Pharm Technology Co. Ltd. (Shanghai, China) according to its standard protocols.

### 2.4. Sequence data processing

Raw sequence data were processed and analyzed using the QIIME software package (Caporaso et al., 2010). The extraction of non-repeated sequences from the optimized sequence was convenient for reducing the redundant calculation in the intermediate process of analysis. Operational taxonomic unit (OTU) clustering was performed according to 97% similarity on non-repeated sequences (excluding single sequence). Chimeras were removed prior to the clustering process to obtain a representative OTU sequence. In order to classify species according to OTU sequences, the RDP classifier Bayesian algorithm was used to classify the representative OTU sequences at a 97% sequence similarity level.

### 2.5. Statistical analysis

The one-way analysis of variance (ANOVA), with the least significant difference (LSD) method, was performed using SPSS 19.0 to determine whether the differences between treatments were significant at the *p* < 0.05 level. The analysis of similarity (ANOSIM) was performed based on the Bray-Curtis distance using the *vegan* package in R (Version 3.3.1). The Mantel test was performed based on Bray-Curtis in QIIME software (Version 1.9.1). QIIME was used to calculate the β diversity distance matrix, while the *vegan* package was used for Nonmetric Multidimensional Scaling (NMDS). The Shannon and Chao1 indices were calculated in R to estimate α-diversity of the soil bacterial and fungal communities. The Phylogenetic diversity (PD) indices were analyzed on the online tool of Majorbio Cloud Platform.^1^ Distance-based redundancy analysis (db-RDA) was performed using the *vegan* package in R. The distance-decay relationships (DDRs) were investigated based on taxonomic dissimilarities (Sorensen distance) (Oksanen et al., 2020).

The slope of the ordinary least-squares regression between the log-transformed geographic distance and microbial community similarity was used to quantify the distance-decay rate of the microbial community using the *vegan* R package (Dixon, 2003). One-way ANOVA was performed to examine the significance of the slopes of the two curves using the *lsmeans* R package (Lenth, 2016). Null modeling-based approaches were performed to infer community assembly mechanisms (Chase and Myers, 2011; Stegen et al., 2013; Chase et al., 2019). First, representative OTU sequences were introduced into MEGA software (Version 10.2.4) to build a phylogenetic tree using the maximum likelihood method, then the *ape* R package was loaded to read the phylogenetic tree (.tre) and OTU-table (.csv) files. The mean nearest phylogenetic taxon distance (MNTD) and nearest taxon index (NTI) indices were calculated using the *Picante* package (Kembel et al., 2010). An average NTI value greater than 0 indicates sample aggregation and the next step calculation can be performed, while an average NTI value lower than 0 indicates sample dispersion requiring further re-grouping. In addition, the βMNTD and βNTI indices of different samples were calculated using the same calculation method. The last operation is the RCbray index (Stegen et al., 2013). The *Picante* package was used to further quantify the deterministic (heterogeneous and homogenous selections) and randomness processes (diffusion limits and co-diffusion). The null model, based on the βNTI and RCbray indices, was applied to quantify the contributions of the deterministic and randomness processes to soil microbial community assembly. βNTI values that were lower than −2 or higher than 2 indicated that the communities were driven by homogeneous or heterogenous processes. In addition, a |βNTI| value lower than 2 suggested a stochastic assembly process of microbial community, and the diffusion limit (RCbray >0.95), isotropic diffusion (RCbray < −0.95), and uncertain process (−0.95 < RCbray <0.95) were determined according to the size of RCbray index in the assembly processes of microbial communities (Stegen et al., 2015).

## 3. Results

### 3.1. Diversity, composition and structure of microbial communities

**Figure 2:**
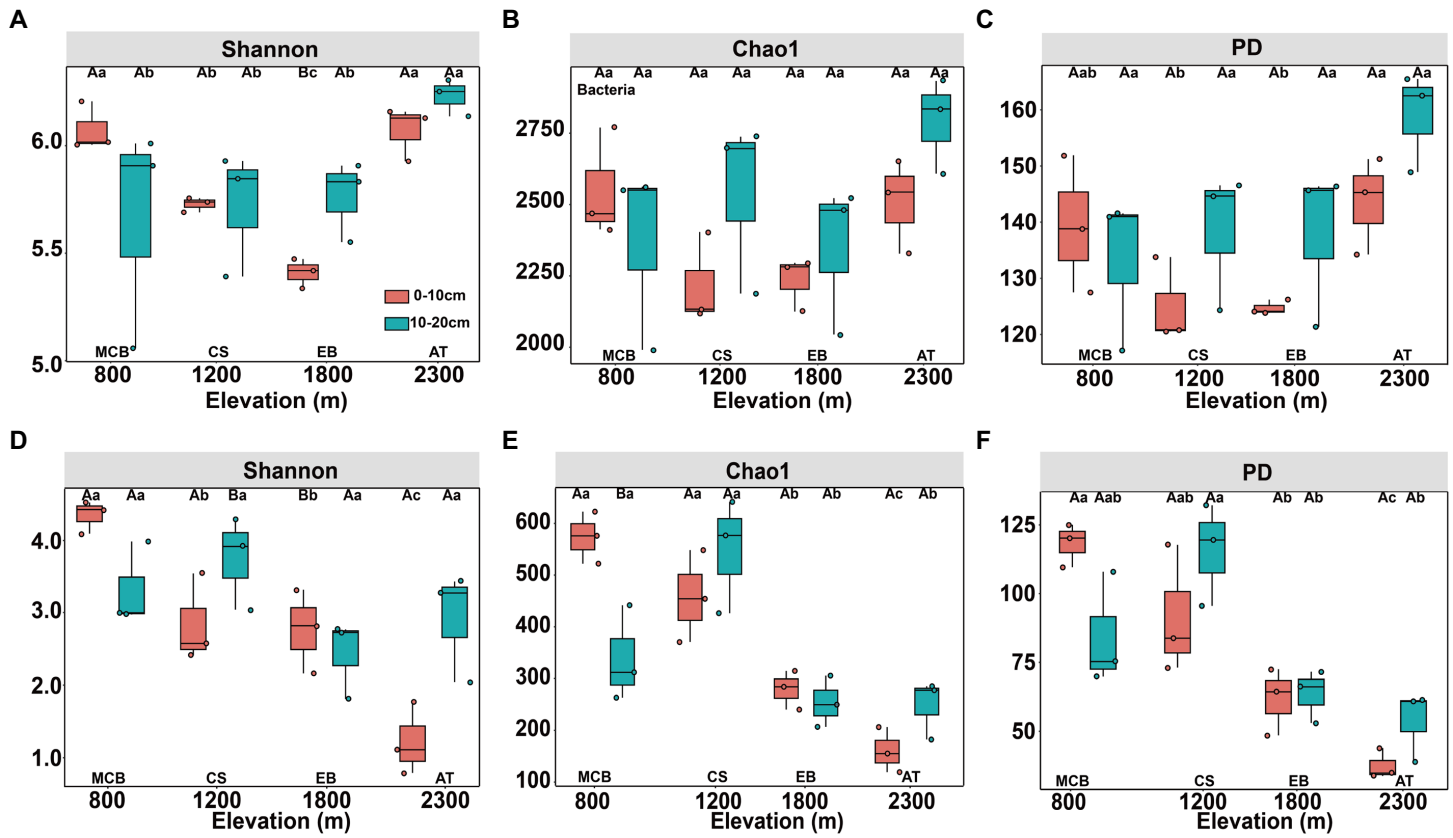
Changes in diversity of soil bacterial **(A–C)** and fungal **(D–F)** communities along altitudinal gradient and soil depth on Changbai Mountain. Capital letters indicate significant differences (*p* < 0.05) between soil depth treatments, lower case letters indicate significant differences (*p* < 0.05) between altitude treatments.

**Figure 3:**
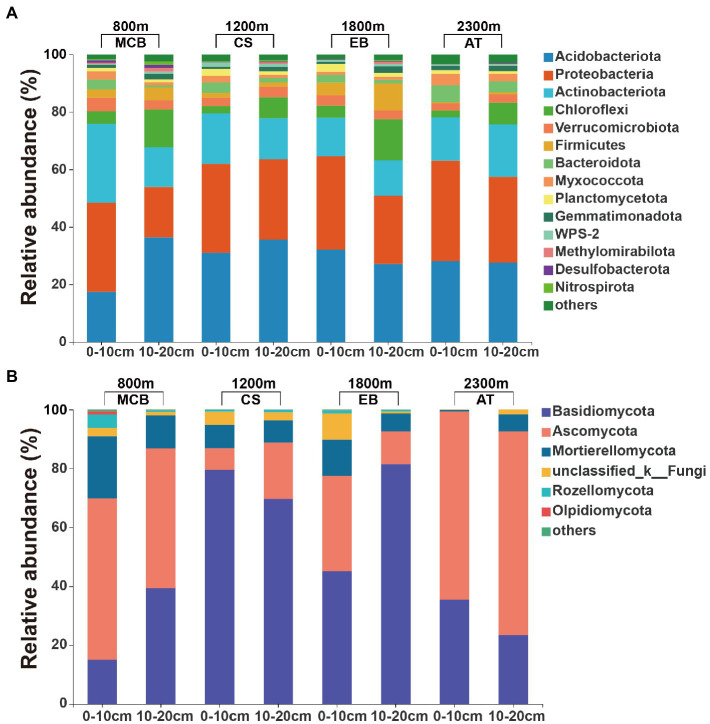
Relative abundances of the major soil **(A)** bacterial and **(B)** fungal communities at the phylum level.

**Figure 4:**
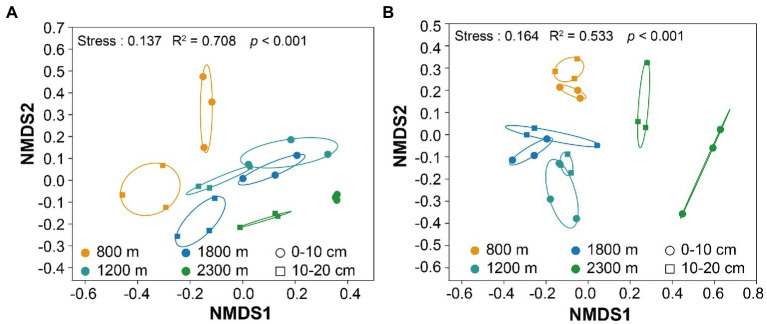
Non-metric multidimensional scaling (NMDS) of the bacterial **(A)** and fungal **(B)** communities along altitudes and soil depths.

The minimal bacterial Shannon, Chao1, and PD diversity indices at 0–10 cm depth were observed at 1800 m, showing a U-shaped altitudinal pattern, while the fungal α-diversity indices decreased with altitude (Figure 2 and Supplementary Figure S1). The bacterial diversity showed no obvious altitude pattern at 10–20 cm depth, while the fungal Chao1 and PD indices showed hump patterns with increasing altitude (Figure 2 and Supplementary Figure S2). The two most abundant bacterial phyla were Acidobacteria and Proteobacteria, accounting for 48.41–64.58% of the pyrosequences, followed in abundance by Actinobacteriota (12.31–27.48%), Chloroflexi (2.34–14.33%), Verrucomicrobiota (2.50–4.78%; Figure 3). The two most abundant fungal phyla were Basidiomycota and Ascomycota, accounting for 69.82–99.37% of the pyrosequences, followed by Mortierellomycota (0.52–21.14%), unclassified_k__Fungi (0.1–8.96%), and Rozellomycota (0.01–4.52%; Figure 3). The relative abundance of Proteobacteria at 0–10 cm depth was significantly higher than that at 10–20 cm soil depth at all altitudes (*F* = 13.77, *p* < 0.05, 800 m; *F* = 18.77, *p* < 0.05, 1800 m; *F* = 15.35, *p* < 0.05, 2300 m), except for 1200 m (Supplementary Figure S3). At 1800 m, the relative abundance of Basidiomycota at 0–10 cm soil depth was significantly lower (*F* = 9.17, *p* < 0.05) than that at 10–20 cm soil depth (Supplementary Figure S3). The NMDS plots showed different soil microbial compositions in the collected soil samples with respect to different altitudes (Figure 4). In addition, both soil depths showed significant variability along the altitude gradient (Figure 4 and Table 1).

**Table 1:** Anosim and Adonis analysis of altitude and soil depth in shaping the bacterial and fungal communities structure.

	Bacteria				Fungi			
	Anosim		Adonis		Anosim		Adonis	
	*F*	*p*	*F*	*p*	*F*	*p*	*F*	*p*
0–10 cm	0.864	<0.001	0.651	<0.001	0.877	<0.001	0.525	<0.001
10–20 cm	0.941	<0.001	0.641	<0.001	0.966	<0.001	0.494	<0.001

### 3.2. Correlations between microbial communities and soil properties

**Figure 5:**
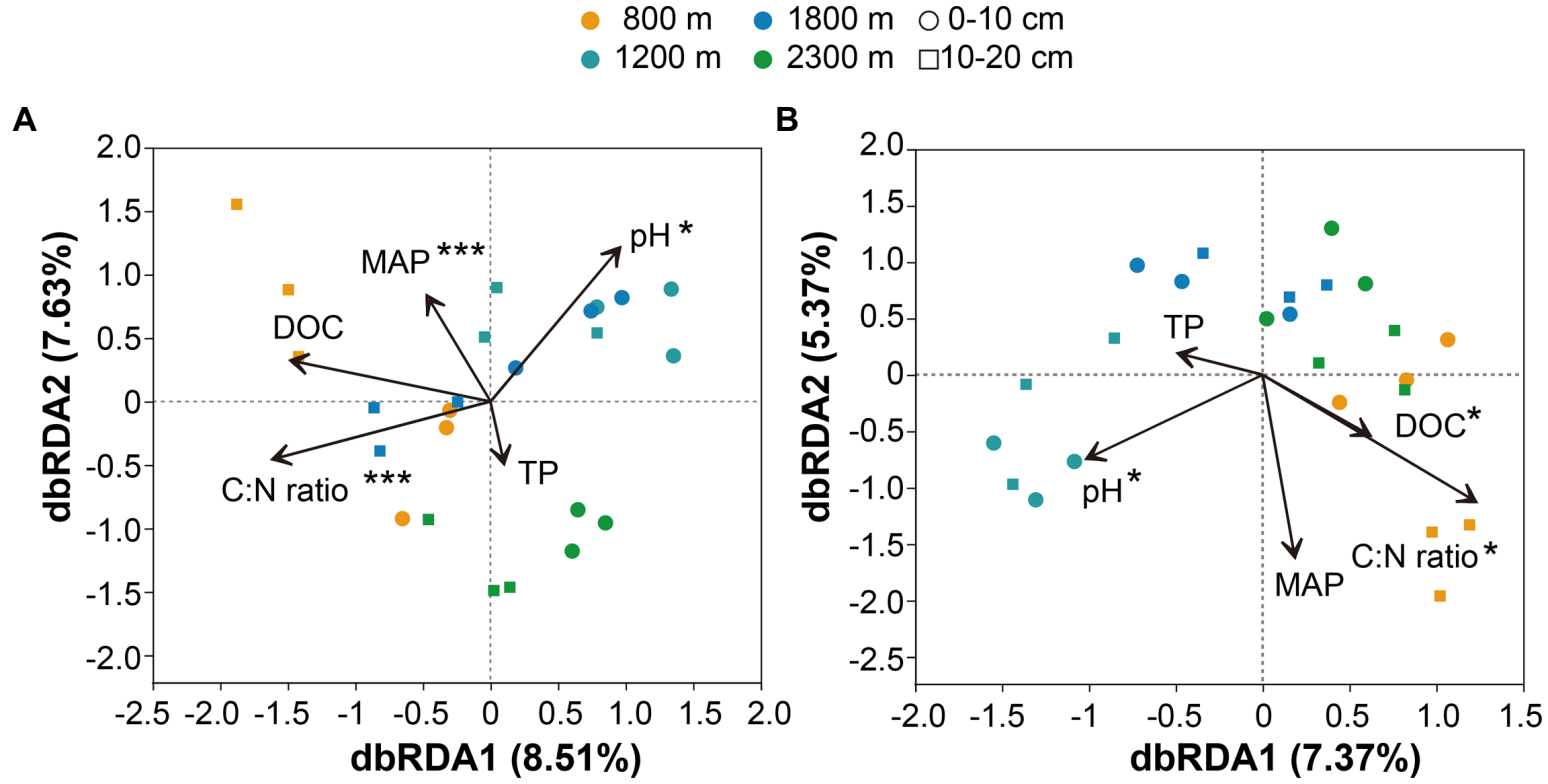
Distance-based redundancy analysis of soil bacterial **(A)** and fungal **(B)** communities along altitudinal gradient and soil depth as related to environmental variables. pH: pondus Hydrogenii; DOC, dissolved organic carbon; TP, total phosphorus; C:N ratio, TC/TN; MAP, mean annual precipitation. *p<0.05; **p<0.01; ***p<0.001.

**Figure 6:**
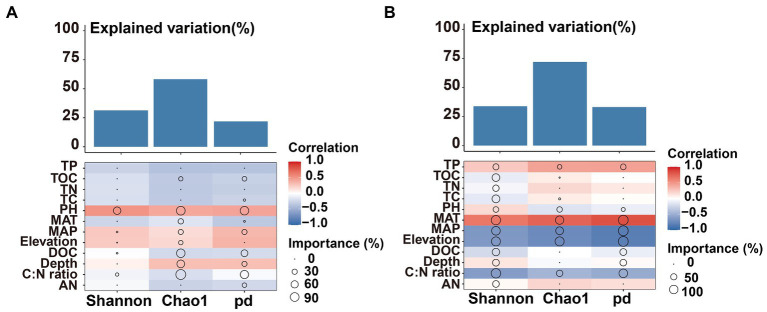
Contributions of environmental factors to bacterial bacterial **(A)** and fungal **(B)** α-diversity based on correlation and random forest model. Circle size represents the variable’s importance (i.e., percentage of increase of mean square error calculated *via* random forest model). Colors represent spearman’s correlations.

The Mental test showed significant correlations between community composition and environmental variables (Table 2). The C:N ratio showed significant positive correlations with the soil bacterial community compositions at 0–10 cm (*r* = 0.757, *p* < 0.001) and 10–20 cm (*r* = 0.387, *p* < 0.05) soil depths (Table 2). Soil TP contents exhibited a significant positive correlation with the soil fungal community composition at 0–10 cm soil depth (*r* = 0.451, *p* < 0.001), while TOC was the most relevant soil variables to the soil fungal community at 10–20 cm soil depth (*r* = 0.341, *p* < 0.01; Table 2). Distance-based redundancy analysis (db-RDA) demonstrated the stronger effects of the soil pH and C:N ratio on the bacterial and fungal communities, respectively (Figure 5). Among the abiotic factors, C:N ratio and soil pH were the two major soil variables contributing to the bacterial α-diversity variation (Figure 6A), while C:N ratio and altitude were the two major soil variables contributing to the fungal α-diversity variations (Figure 6B).

**Table 2:** Results of Mantel test on the correlation between community composition (Bray-Curtis) and environmental variables for bacteria and fungi.

Variable	Bacteria				Fungi			
	0–10 cm		10–20 cm		0–10 cm		10–20 cm	
	*r*	*p*	*r*	*p*	*r*	*p*	*r*	*p*
TP	**0.387**	**0.014**	−0.035	0.812	**0.451**	**0.001**	**0.214**	**0.027**
DOC	**0.274**	**0.050**	0.210	0.114	0.101	0.359	**0.258**	**0.027**
AN	0.244	0.064	0.066	0.648	**0.378**	**0.005**	0.022	0.825
pH	**0.301**	**0.021**	0.124	0.313	**0.279**	**0.018**	**0.261**	**0.022**
TN	**0.292**	**0.035**	−0.022	0.866	**0.369**	**0.003**	0.163	0.130
TC	**0.325**	**0.031**	0.258	0.051	0.172	0.154	**0.320**	**0.011**
TOC	**0.264**	**0.037**	**0.301**	**0.028**	0.097	0.413	**0.341**	**0.007**
C:N ratio	**0.757**	**0.001**	**0.387**	**0.015**	**0.350**	**0.005**	**0.339**	**0.008**
MAT	**0.605**	**0.001**	**0.632**	**0.001**	**0.630**	**0.001**	**0.590**	**0.001**
MAP	**0.575**	**0.008**	**0.674**	**0.001**	**0.622**	**0.001**	**0.625**	**0.001**
Altitude	**0.668**	**0.001**	**0.697**	**0.001**	**0.545**	**0.001**	**0.606**	**0.001**

r, correlation coefficient of environmental factors with microbial communities; *p*-value, statistical significance; values in bold indicate significant correlations (*p* < 0.05). TP, total phosphorus; DOC, dissolved organic carbon; AN, available nitrogen; pH: pondus Hydrogenii; TN, total nitrogen; TC, total carbon; TOC, total organic carbon; C:N ratio, TC:TN; MAT: mean annual temperature; MAP: mean annual precipitation.

### 3.3. Assembly processes and driving factors for microbial communities

**Figure 7:**
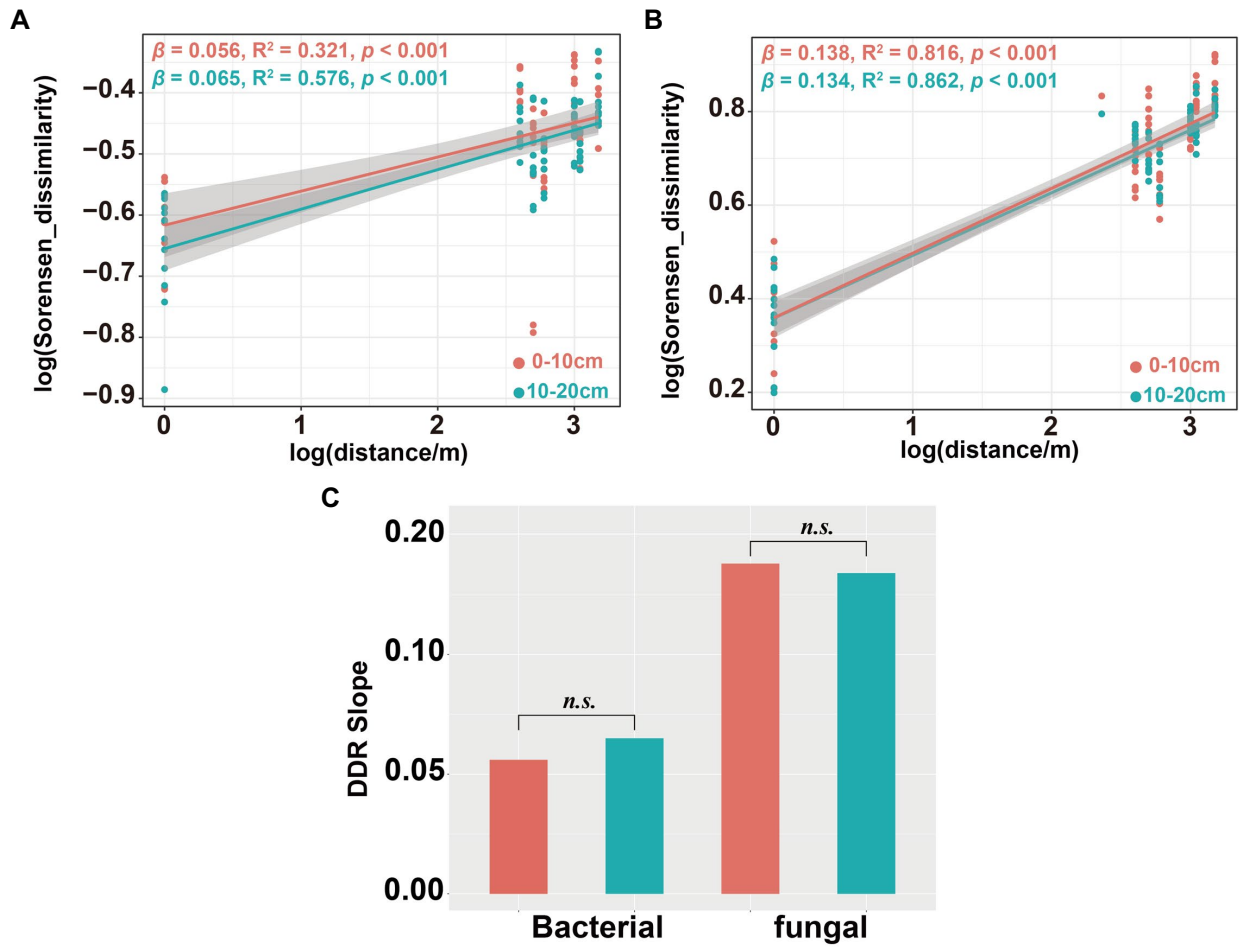
Distance-decay relationship (DDR) for the soil bacterial and fungal communities. DDR based on Sorensen dissimilarity along geographic distances for the bacterial community **(A)**; DDR for the fungal community **(B)** and DDR slope significance test of soil bacterial and fungal communities **(C)**. The slope β of DDR represent the spatial turnover rate of species.

**Figure 8:**
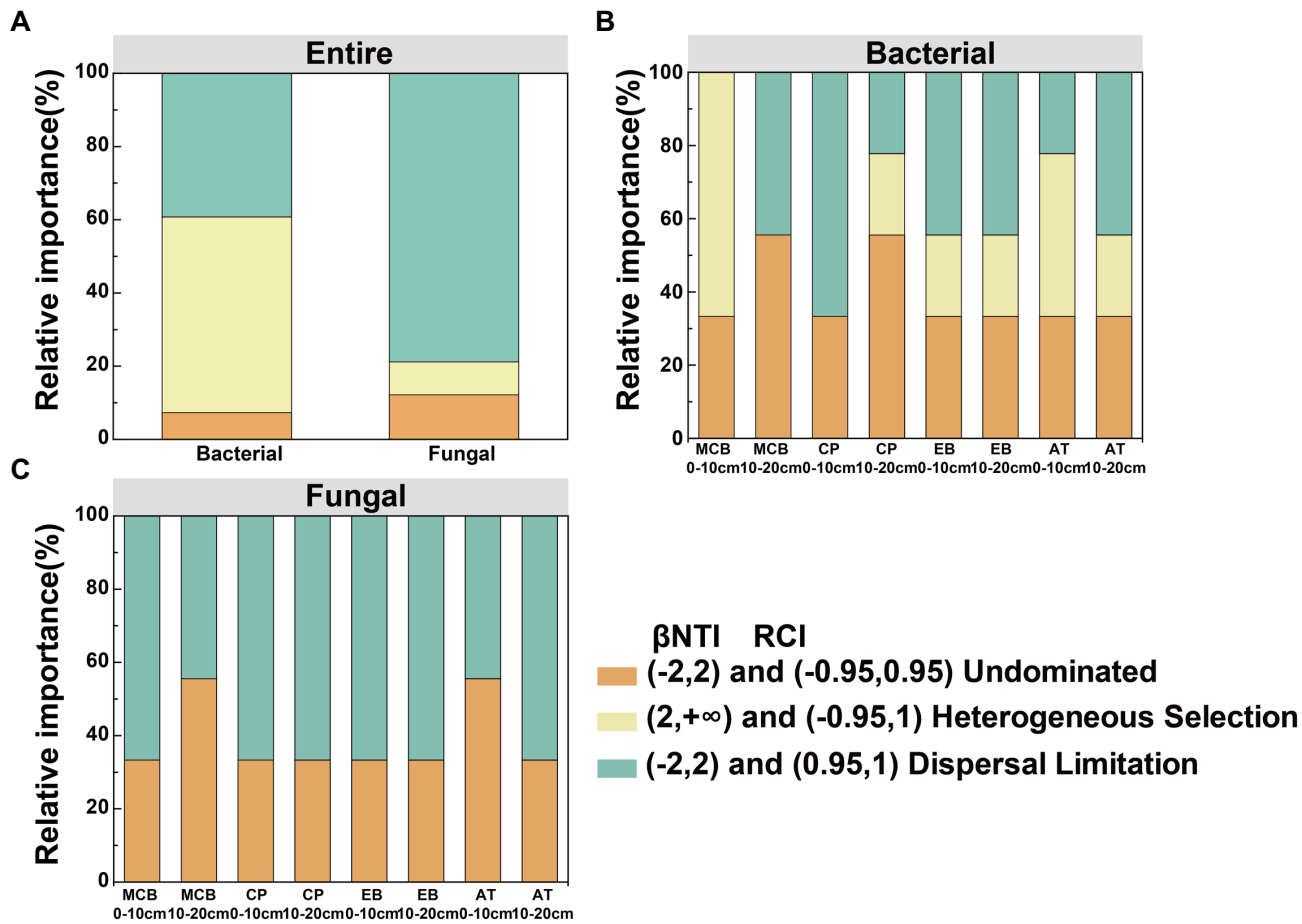
Deterministic and stochastic processes in the assembly of soil bacterial and fungal communities in the Changbai Mountain. These two processes were both detected using the β-Nearest Taxon Index (βNTI) and Raup-Crick Index (RCI). **(A)** Relative influences of deterministic and stochastic assembly processes in shaping a bacterial and fungal community. Relative influences of deterministic and stochastic assembly processes in shaping bacterial community **(B)** and fungal community **(C)** for different treatments.

Community dissimilarity and geographic distance for each pairwise sample set showed a significant distance-decay relationship (DDR) of the microbial communities (Figures 7A,B). At the same soil depth, the spatial turnover rate of fungal community (*β* = 0.134–0.138) was higher than that of bacterial community (*β* = 0.056–0.065; Figures 7A,B). However, there was no significant difference in the turnover rates of soil bacterial and fungal communities between different soil depths (Figure 7C). Bacterial community assembly was determined by deterministic process (53.47%), while assembly processes of soil fungal communities was mainly determined by stochastic process (90.62%; Figure 8A). At 0–10 cm depth, the effect of the deterministic process (heterogeneous selection) on soil bacterial community assembly was dominated at the lowest altitude (800 m), while that of the stochastic process (undominated and dispersal limitation) exhibited a gradual dominance at the remaining altitudes (Figure 8B). The soil bacterial community assembly was mostly driven by the stochastic process at 10–20 cm soil depth (Figure 8B). In contrast, the fungal community assembly was dominated by the stochastic process among all selected altitudes and soil depths (Figure 8C).

**Figure 9:**
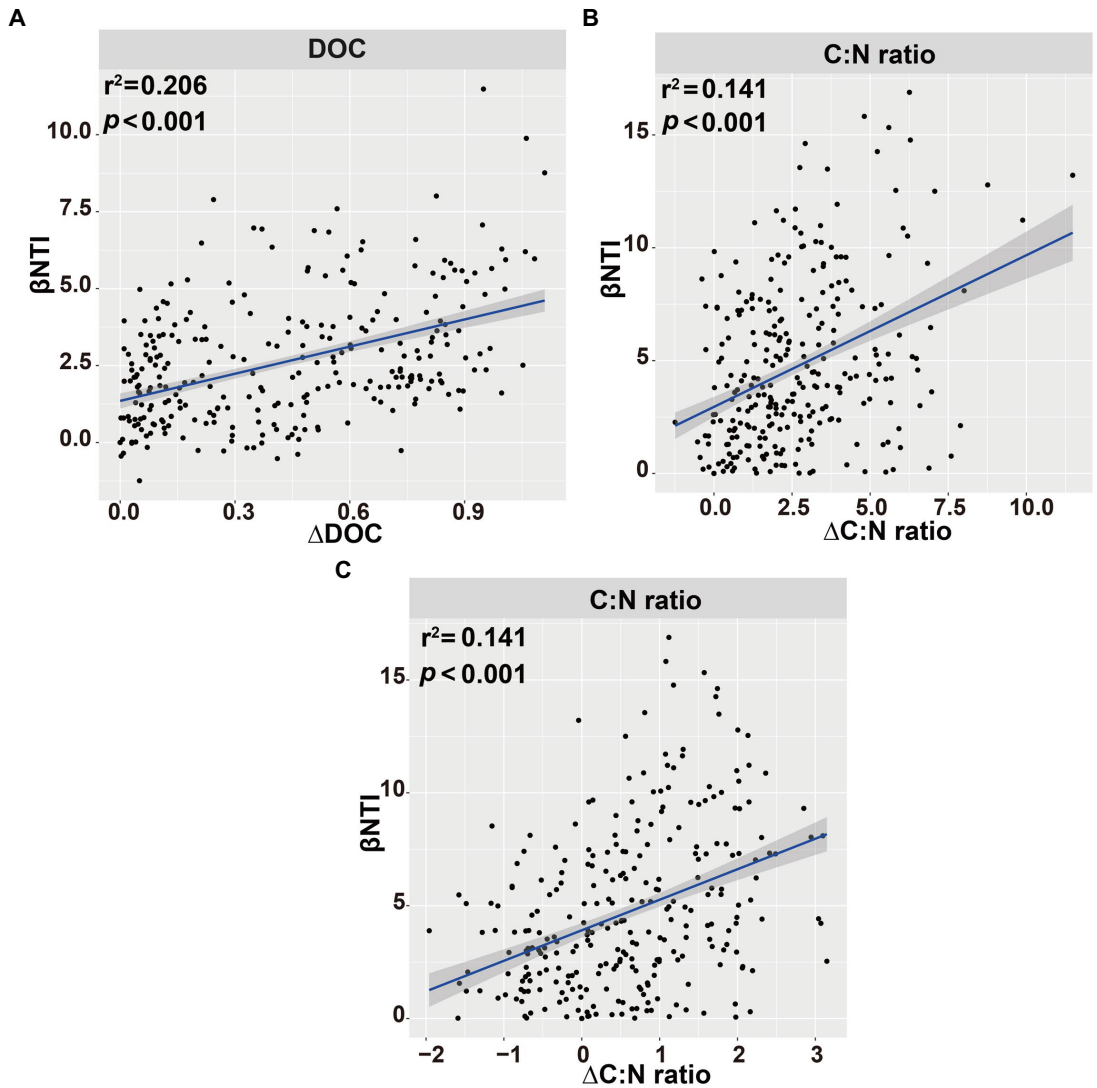
Linear regression analysis of the connections between β-nearest taxon index (βNTI) of soil bacterial **(A,B)** or fungal **(C)** communities and soil variables. Blue shaded area indicates 95% confidence interval (Spearman’s *p* < 0.05).

The main explanatory soil variables were selected to determine the correlations between the assembly processes of microbial communities and environmental variables (based on Euclidean distance matrices; Table 3). For bacterial community, the significant positive correlations of βNTI with DOC (*r*^2^ = 0.206, *p* < 0.001) and C:N ratio (*r*^2^ = 0.141, *p* < 0.001; Figure 9). Whereas for fungal community, a significant positive correlation was observed between the soil C:N ratio and βNTI (*r*^2^ = 0.141, *p* < 0.001; Figure 9).

**Table 3:** Mantel tests of environmental variables against the phylogenetic turnover (β-nearest taxon index) of the soil bacterial and fungal communities.

Variable	Bacteria		Fungi	
	Mantel r	*p*	Mantel r	*p*
TP	−0.0380	0.9952	0.0057	0.2145
DOC	0.0408	0.0004	0.0131	0.0082
AN	0.0054	0.3130	0.0082	0.0740
pH	0.0048	0.3630	0.0355	0.0001
TN	−0.0100	0.7858	−0.0017	0.5988
TC	−0.0098	0.7484	−0.0048	0.7798
TOC	−0.0140	0.8465	−0.0020	0.6018
C:N ratio	0.0306	0.0354	0.1039	0.0001
MAP	0.0443	0.0680	−0.0054	0.6798
MAT	0.0478	0.0456	−0.0059	0.6886
Altitude	−0.0129	0.8735	0.0003	0.4556

TP, total phosphorus; DOC, dissolved organic carbon; AN, available nitrogen; pH: pondus Hydrogenii; TN, total nitrogen; TC, total carbon; TOC, total organic carbon; C:N ratio, TC:TN; MAT: mean annual temperature; MAP: mean annual precipitation.

The multiple regression model (MRM) was used to further assess the relative contributions of each environmental factor to the soil bacterial and fungal communities (Table 4). According to the obtained results, the soil C:N ratio (*R*^2^ = 0.419, *p* < 0.001) and altitude (*R*^2^ = 0.534, *p* < 0.001) significantly explained the variations of soil bacterial community dissimilarities at the upper and deeper soil depths, respectively (Table 4). Whereas MAP significantly explained the variations in the soil fungal community dissimilarities at both soil depths (Table 4).

**Table 4:** Results of the multiple regression analysis on matrices analysis (MRM) for the microbial community composition.

	Bacteria				Fungi			
Variable	0–10 cm		10–20 cm		0–10 cm		10–20 cm	
	*R* ^2^	*p*	*R* ^2^	*p*	*R* ^2^	*p*	*R* ^2^	*p*
TP	0.132	0.036	0.004	0.698	0.189	0.003	0.034	0.103
DOC	0.044	0.099	0.027	0.225	0.015	0.405	0.028	0.154
AN	0.094	0.046	0.059	0.092	0.390	<0.001	0.019	0.222
pH	0.161	0.006	0.005	0.590	0.009	0.435	0.045	0.066
TN	0.058	0.109	0.012	0.451	0.435	0.002	0.009	0.387
TC	0.102	0.102	0.011	0.456	0.043	0.116	0.046	0.066
TOC	0.079	0.040	0.014	0.376	0.018	0.344	0.051	0.055
C:N ratio	0.419	<0.001	0.215	0.005	0.247	0.002	0.106	0.015
MAP	0.254	0.005	0.424	<0.001	0.711	<0.001	0.531	<0.001
MAT	0.335	0.004	0.394	<0.001	0.668	<0.001	0.480	<0.001
Altitude	0.346	<0.001	0.534	<0.001	0.437	<0.001	0.521	<0.001

The proportion of variation in Sorensen dissimilarity matrices is explained by the remaining variables.TP, total phosphorus; DOC, dissolved organic carbon; AN, available nitrogen; pH: pondus Hydrogenii; TN, total nitrogen; TC, total carbon; TOC, total organic carbon; C:N ratio, TC:TN; MAT, mean annual temperature; MAP, mean annual precipitation.

## 4. Discussion

The altitudinal diversity pattern is one of the most fundamental patterns in animal and plant biogeography (Denef et al., 2009). Here we observed that soil bacterial diversity appeared a U-shaped pattern with altitude at 0–10 cm soil depth, which is in line with the previous results (Singh et al., 2014). The α-diversity of soil bacterial communities did not show an obvious distribution pattern with altitudes at 10–20 cm soil depth, which was consistent with the results revealed in previous studies (Fierer et al., 2011). The peak pattern (Miyamoto et al., 2014; Wang et al., 2015; Ogwu et al., 2019) or U-shaped pattern (Liu et al., 2018) of soil fungal community were observed in previous studies, showing the highest and lowest diversity observed at mid-altitude. The α-diversity of fungal community at 0–10 cm soil depth exhibited a monotonically decreasing trend with altitude (Figure 2 and Supplementary Figure S2), while the Chao1 and PD indices of the fungal communities at 10–20 cm soil depth showed a hump-shaped pattern (Figure 2 and Supplementary Figure S3). In addition, the significant differences of ectomycorrhizal fungi distribution between soil depths might resulted in different substrate utilization patterns (Dickie et al., 2002).

Both bacterial and fungal communities were significantly different among the selected altitudes and soil depth (Figure 4 and Table 1). We found bacterial and fungal communities have significant correlation with altitude (Table 2). Previous research found that bacterial community composition varied with elevation (Fierer et al., 2011). The fungal community composition had significant differences along the altitude gradient (Kivlin et al., 2014), which might be due to the different selection of soil habitats by different fungal groups, with obvious ecological niche differentiation (Cook and Quinn, 1995). It had been reported that the spatial distribution pattern of fungal community was closely related to environmental heterogeneity (pH, soil nutrition and altitude) (Cox et al., 2010; Polme et al., 2013). Acidobacteria and Proteobacteria were the major soil microorganisms on Changbai Mountain (Figure 3), which was consistent with the findings reported in previous studies (Shen et al., 2013). This might be due to the crucial role of Acidobacteria in organic matter decomposition and nutrient cycling (Eichorst et al., 2018). Proteobacteria have high morphological and metabolic diversity, which enables them to easily utilize soil organic carbon and nitrogen (Bryant and Frigaard, 2006; Mukhopadhya et al., 2012). Basidiomycetes and Ascomycetes were the dominant soil fungal groups due to the presence of a thicker litter layer in the upper soil layer than in the deeper soil layer. Indeed, Basidiomycetes can decompose complex lignocellulosic components (Lundell et al., 2010). Ascomycetes can use more resources to better tolerate environmental pressure (Yang et al., 2017; Egidi et al., 2019).

Soil nitrogen (N) and phosphorus (P) are important nutrients for bacterial growth and activity, influencing the spatial distribution of soil microorganisms (Wang et al., 2018; Zhang et al., 2019). Soil fungal diversity in forests was related to plant diversity, temperature, and altitude (Geml et al., 2014; Wang et al., 2015; Wan and He, 2020). Previous studies have pointed out that soil pH was a main factor affected the distribution pattern of soil microbial communities along altitudinal gradients (Baath and Anderson, 2003; Lauber et al., 2009; Delgado-Baquerizo et al., 2017). Climatic factors (MAT and MAP) were dominant variables affected the diversity and community composition of bacteria (Singh et al., 2014; Xu et al., 2014; Nottingham et al., 2018; Frindte et al., 2019). The results of this study showed significant positive correlations of TP, DOC, pH, TN, TC, TOC, C:N ratio, MAT, MAP and altitudes with the bacterial community composition at upper soil depth, while TOC, C:N ratio, MAT, MAP and altitudes exhibited significant positive correlations with the bacterial community at deeper soil depth (Table 2). It is noteworthy that the soil fungal community composition exhibited significant positive correlations with soil TP and TOC contents at the upper and deeper soil depths, respectively (Table 2). Soil pH leads to niche differentiation of soil microbial and controls their patterns along altitude gradients (Shen et al., 2013; Bahram et al., 2018). Whereas soil C:N ratio and phosphorus contents could significantly affect the soil fungal diversity in forests and farmland (Lauber et al., 2013; Shen et al., 2017; Li et al., 2018; Delgado-Baquerizo and Eldridge, 2019). The importance of MAT had been widely reported at both horizontal and vertical scales (Yang et al., 2014; Delgado-Baquerizo et al., 2016; Zhou et al., 2016). Among the abiotic factors considered in this study, soil pH and C:N ratio were the major factors contributing to the variation in the soil bacterial α-diversity (Figure 6A), which is consistent with the results observed in Southwest Tibet (Peay et al., 2017; Shen et al., 2019), indicating that soil pH was the main factor controlling soil microbial diversity. Besides soil pH, soil C:N ratio and altitude showed correlations with microbial diversity (Singh et al., 2014; Shen et al., 2017; Li et al., 2018; Delgado-Baquerizo and Eldridge, 2019). This study demonstrated the contributions of the soil C:N ratio and altitudes to the variations in the fungal α-diversity (Figure 6B).

Several studies have shown that soil microbial community have obvious spatial scale patterns in DDRs (Soininen et al., 2007; Morlon et al., 2008; Deng et al., 2016). We observed steeper turnover slopes in the fungal community (*β* = 0.134–0.138) than that in the bacterial community (*β* = 0.056–0.065). This indicates greater dissimilarity of the soil fungal community over geographic distance than that of the soil bacterial community (Figures 7A,B). In addition, we also observed significant DDR for both bacterial (0–10 cm: *R*^2^ = 0.321, *p* < 0.001;10–20 cm: *R*^2^ = 0.576, *p* < 0.001) and fungal (0–10 cm: *R*^2^ = 0.816, *p* < 0.001;10–20 cm: *R*^2^ = 0.862, *p* < 0.001) communities, but soil depth had no significant effects on spatial turnover rate of either bacterial or fungal communities (Figure 7C). These spatial turnover patterns might be due to vegetation characteristics, geographic distance, and environmental conditions (Noss, 1990; Whittaker et al., 2001). Several studies have highlighted two primary factors that alter the spatial turnover rates of microorganisms (Deng et al., 2016). The first factor was niche selection which affects soil microorganism species through environmental heterogeneity, leading to variations in the community composition (Bell, 2010). The second factor was the restriction of spatial microbial dispersion (Cho and Tiedje, 2000; Peay et al., 2010), resulting in disparate soil microbial community (Zhou and Ning, 2017).

Quantifying the relative contributions of the microbial community to its construction provides an understanding of the spatiotemporal distribution patterns of the microbial community (Stegen et al., 2012). Our observation that deterministic assembly in the soil bacterial community was the dominant process (53.47%) and random assembly contributed substantially to the soil fungal community (90.62%; Figure 8), which was in line with the findings of previous studies (Zhang et al., 2018; Liu et al., 2020). This pointed out that soil bacterial community assembly was mainly driven by a deterministic process. Soil fungi require external forces to reproduce, such as wind, underground mycelial proliferation, plant transplantation, and animal activities (Liu et al., 2020).

It is essential to reveal the factors that lead to the dominance of different microbial community assembly processes. Previous studies have pointed out that assembly processes were related to soil chemical properties, such as pH, and NH_4_^+^-N (Jiang et al., 2019; Wan et al., 2021). In this study, the βNTI of bacteria was associated with the soil DOC contents and C:N ratio, while that of fungi was associated with the soil C:N ratio, suggesting that the assembly processes of soil bacterial and fungal communities were affected by soil parameters (Figure 9). Soil organic carbon and nitrogen contents could be readily absorbed by Proteobacteria (Bryant and Frigaard, 2006; Mukhopadhya et al., 2012). The relative abundance of Bacteroidetes was positively correlated with resource availability, which could preferentially consume soil active organic carbon (Fierer et al., 2007).

## 5. Conclusion

Our study revealed changes in the soil microbial community along the altitudinal gradient and soil depth on Changbai Mountain, as well as the main mechanisms controlling the soil microbial community assembly. The obtained results revealed significant differences in the diversity, community composition, and structure of soil microorganisms between altitudes and soil depths. The soil bacterial and fungal communities exhibited clear spatial patterns without showing differences in their spatial turnover rates between soil depths. The community assembly of soil bacteria and fungi were dominated by deterministic and stochastic processes, respectively. The assembly processes of soil bacterial and fungal communities differed along the altitude gradient and soil depth, which were due to the spatial variability in soil factors that control the aggregation of bacterial and fungal communities.

## Data availability statement

The data presented in the study are deposited in the National Center for Biotechnology Information (NCBI) repository, accession number PRJNA936073.

## Author contributions

YK and HW designed the study, conducted the statistical analysis, and wrote the manuscript. YK, YZ, QW, QG, KL, and YL performed the field investigation and collected the data. All authors contributed to the article and approved the submitted version.

## Funding

This work was supported by the National Natural Science Foundation of China (U20A2083), the National Key R&D Program of China (2022YFF1300900), and Jilin Scientific and Technological Development Program (20210509037RQ).

## Conflict of interest

The authors declare that the research was conducted in the absence of any commercial or financial relationships that could be construed as a potential conflict of interest.

## Publisher’s note

All claims expressed in this article are solely those of the authors and do not necessarily represent those of their affiliated organizations, or those of the publisher, the editors and the reviewers. Any product that may be evaluated in this article, or claim that may be made by its manufacturer, is not guaranteed or endorsed by the publisher.

## Footnotes

^1^ https://cloud.majorbio.com/page/tools/
