# Supplementary material for: Differential distribution patterns and assembly processes of soil microbial communities under contrasting vegetation types at distinctive altitudes in the Changbai Mountain
Source: Front Microbiol. 2023 Jun 2;14:1152818. doi: 10.3389/fmicb.2023.1152818 (PMC10272400; doi:10.3389/fmicb.2023.1152818)
Supplement: Supplementary file 1 [file Data_Sheet_1.docx]

Supplementary Material

**Differential distribution patterns and assembly processes of soil microbial communities under contrasting vegetation types at distinctive altitudes in the Changbai Mountain**

**Yujuan Kang^1,2^, Haitao Wu^1*^, Yifan Zhang^1,3^, Qiong Wu^1,3^, Qiang Guan**^1^**, Kangle Lu**^1^**, Yilin Lin**^1^

^1^ Key Laboratory of Wetland Ecology and Environment, Northeast Institute of Geography and Agroecology, Chinese Academy of Sciences, Changchun 130102, China

^2^ University of Chinese Academy of Sciences, Beijing 100049, China

^3^ Jilin Normal University, Jilin 136000, China

* Correspondence: Haitao Wu: wuhaitao@iga.ac.cn

**Supplementary Figures and Tables**

**Supplementary Table 1.** Climate and vegetation characteristics at sampling sites in the Changbai Mountain.

| Altitude (m) | Community type | MAT (℃) | MAP  (mm) | Dominant tree species |
| --- | --- | --- | --- | --- |
| 800 | Mixed coniferous broad-leaved forest | 3.15 | 655 | *Pinus koraiensis, Picea koraiensis, Fraxinus mandshurica, Acer mono, Tilia amurensis, Ulmus davidiana var. japonica, Quercus mongolia, Larix olgensis* |
| 1200 | Coniferous spruce forest | 1.50 | 709 | *Picea jezoensis var. microsperma, Picea koraiensis* |
| 1800 | Ermans birch forest | 0.29 | 751 | *Betula ermanii* Cham |
| 2300 | Alpine tundra | -3.03 | 911 | *Rhododendron aureum, Vaccinium uliginosum Rhododendron redowskianum* Maxim*., Rhododendron confertissimum., Dryas octopetala var.asiatica* |


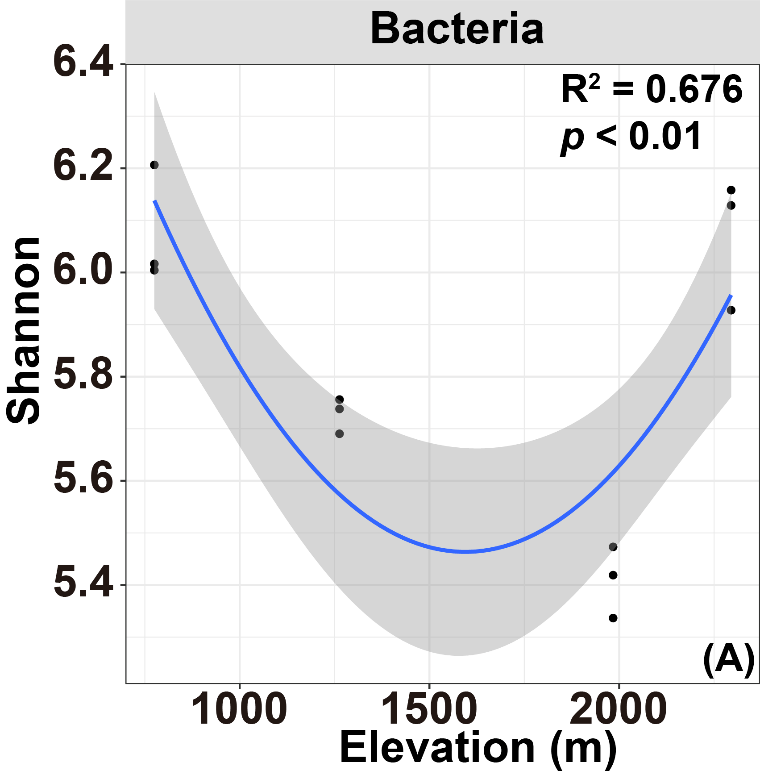

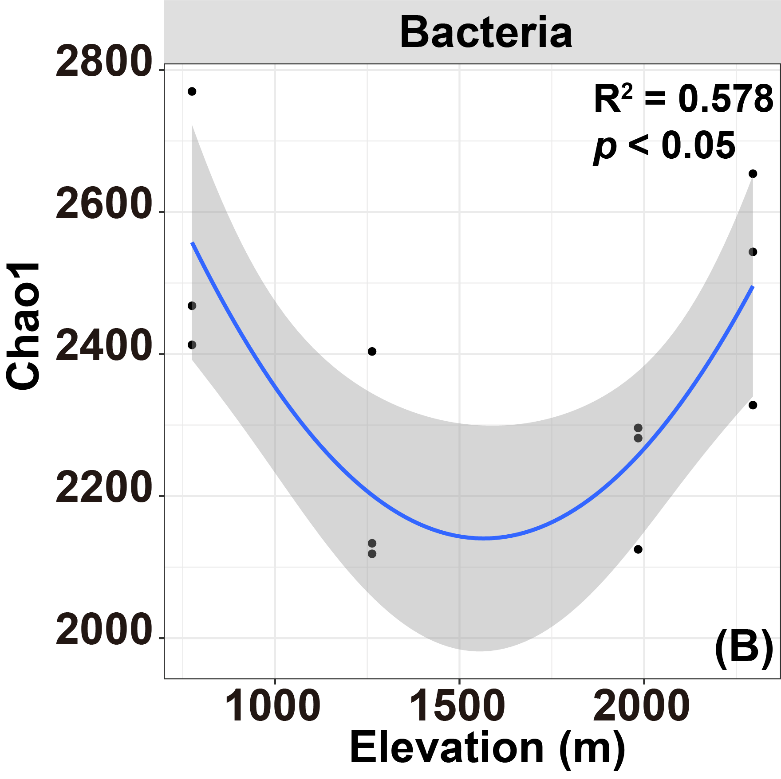


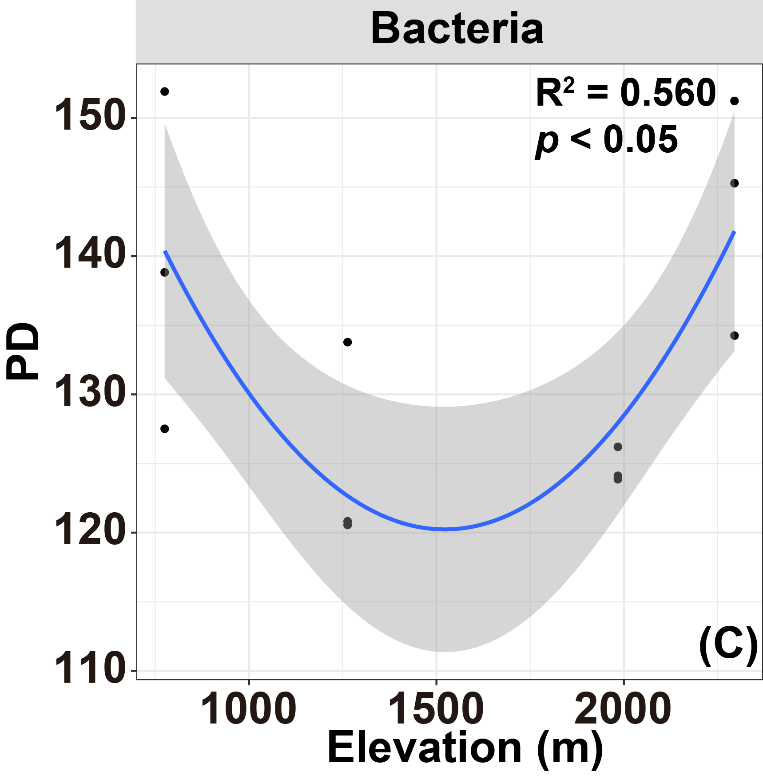

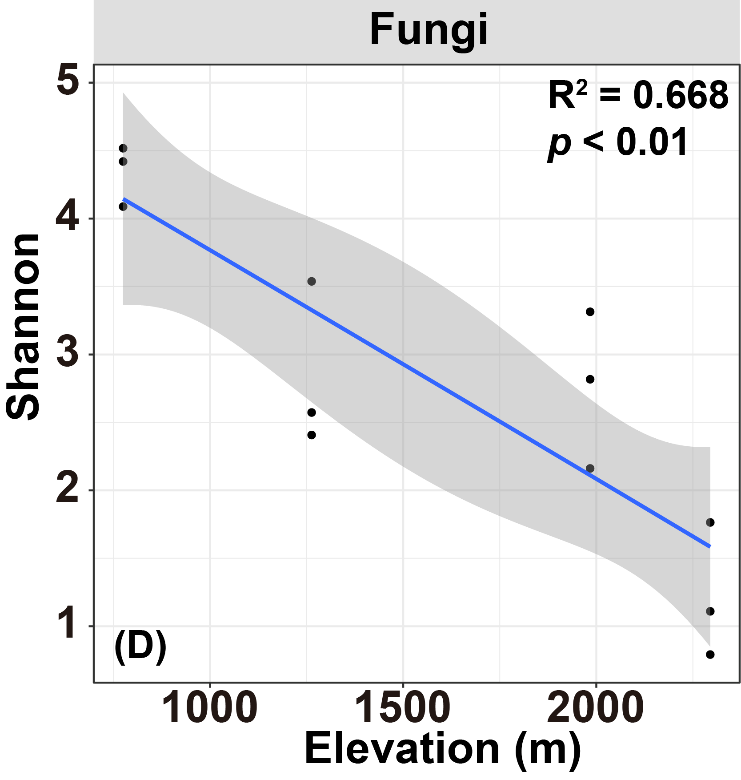

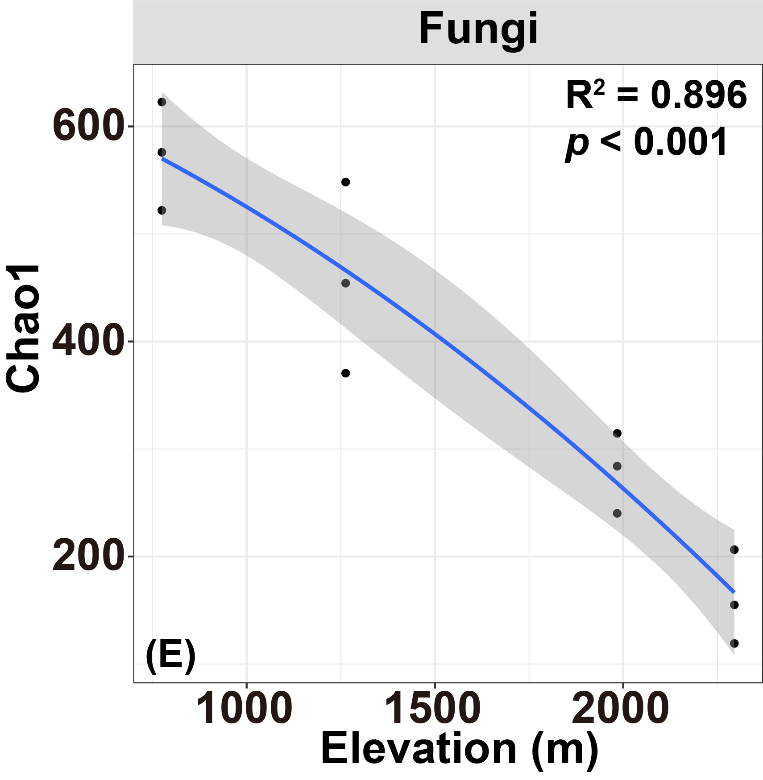

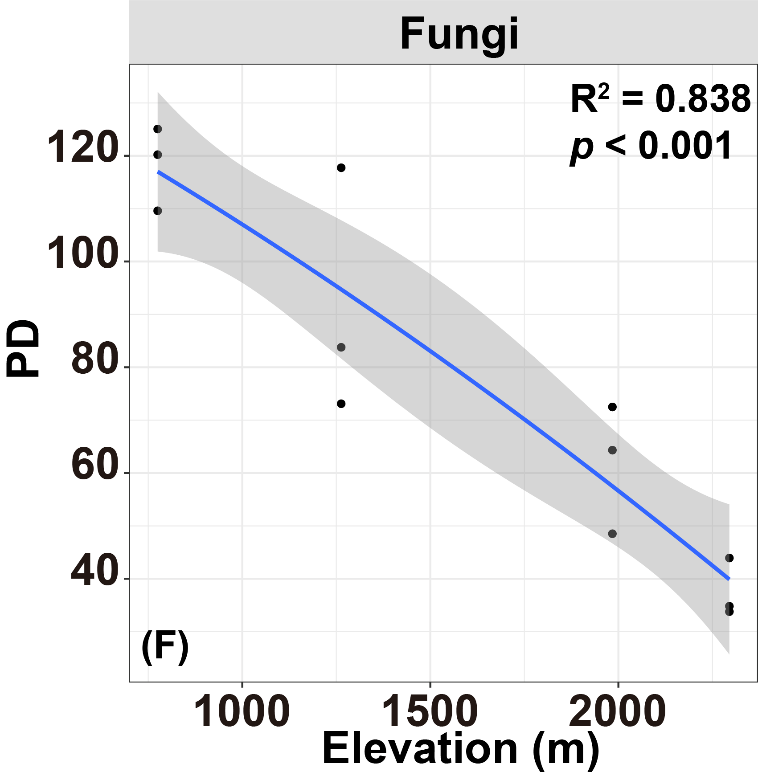


**Supplementary Figure 1.** Shannon, Chao1, and PD indices for bacterial (A, B, C) and fungal (D, E, F) communities and their relationships with altitude at 0-10 soil depths. The relationships were described by quadratic models. p-values indicate significance levels.


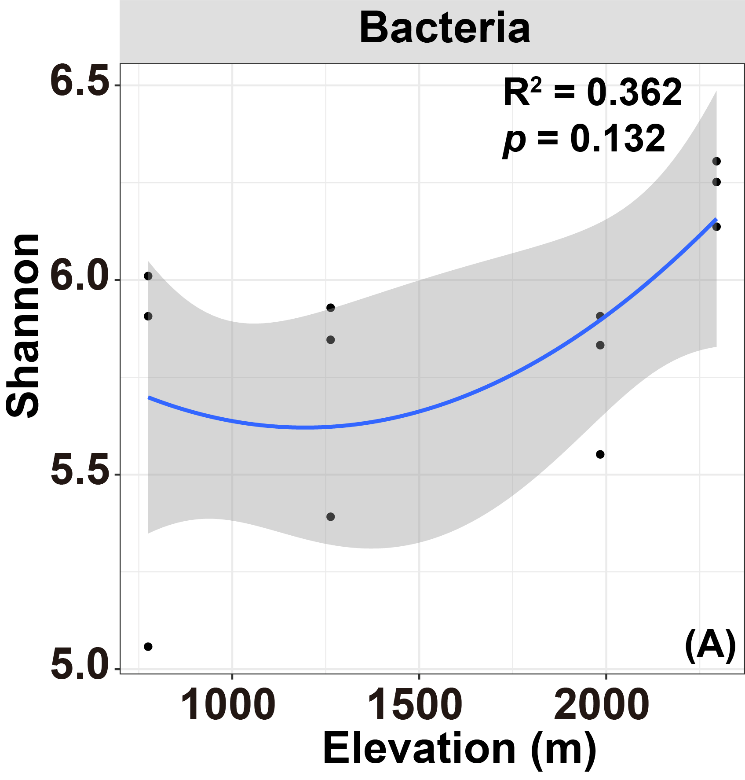

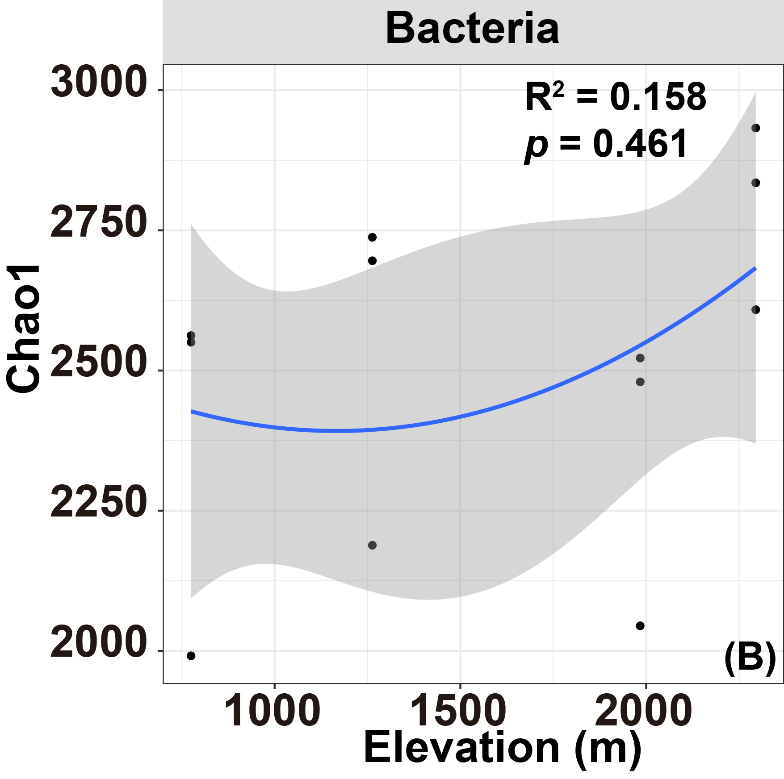


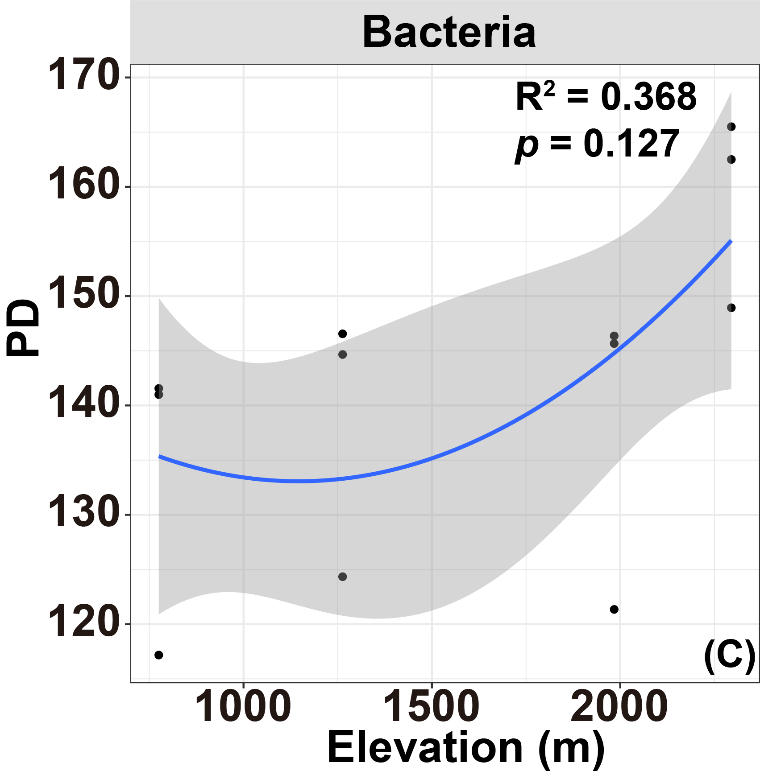

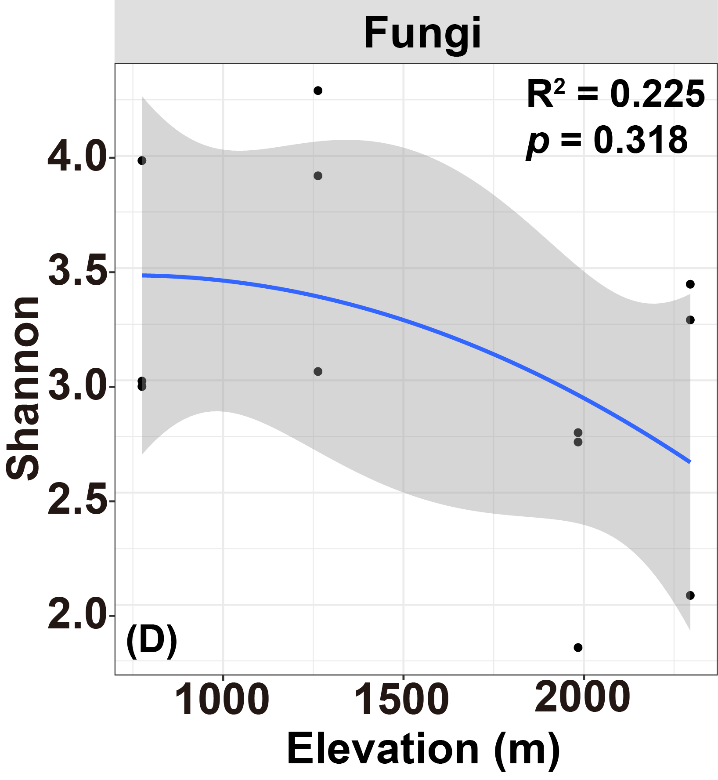


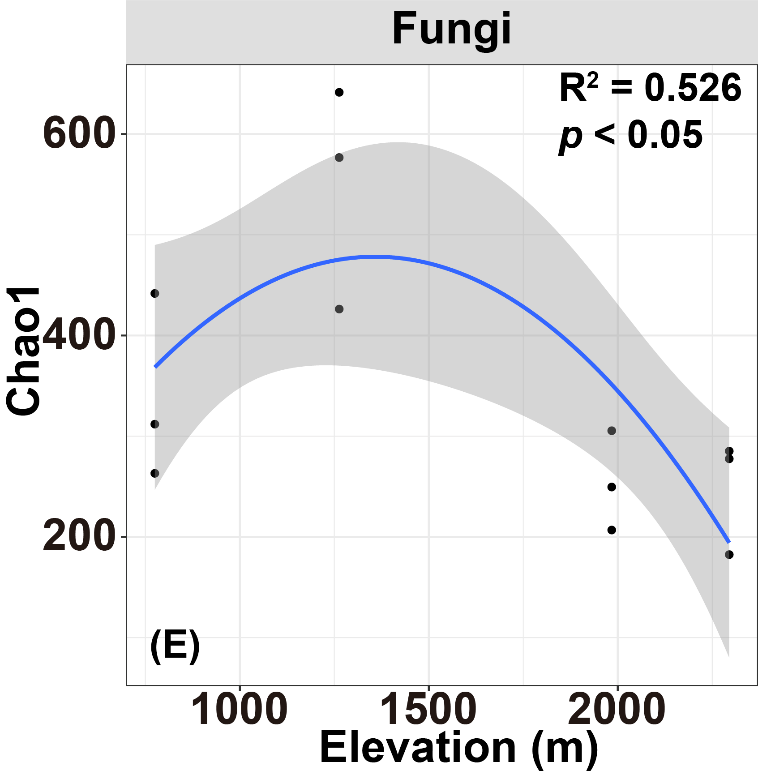

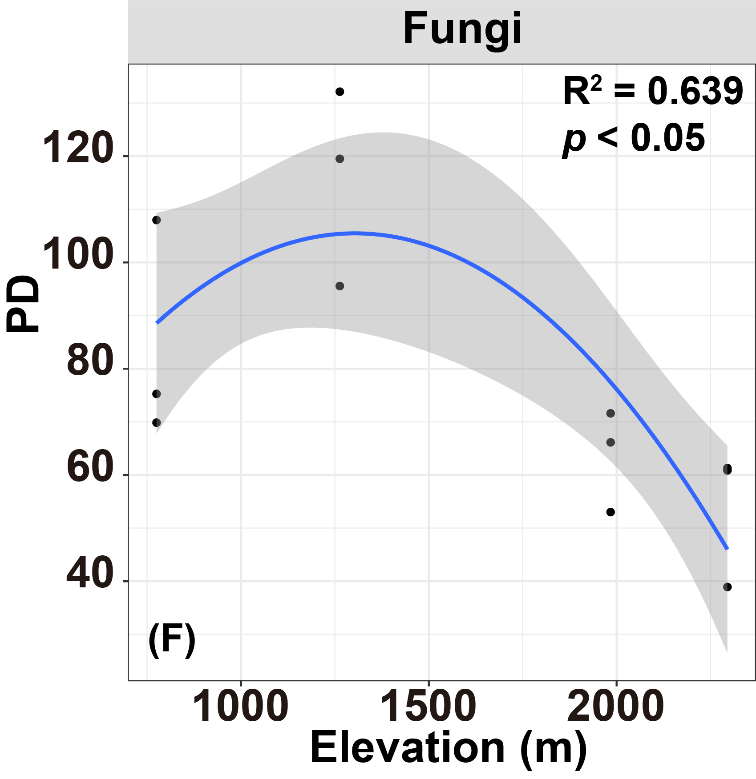


**Supplementary Figure 2.** Shannon, Chao1, and PD indices for bacterial (A, B, C) and fungal (D, E, F) communities and their relationships with altitude at 10-20 soil depths. The relationships were described by quadratic models. p-values indicate significance levels.


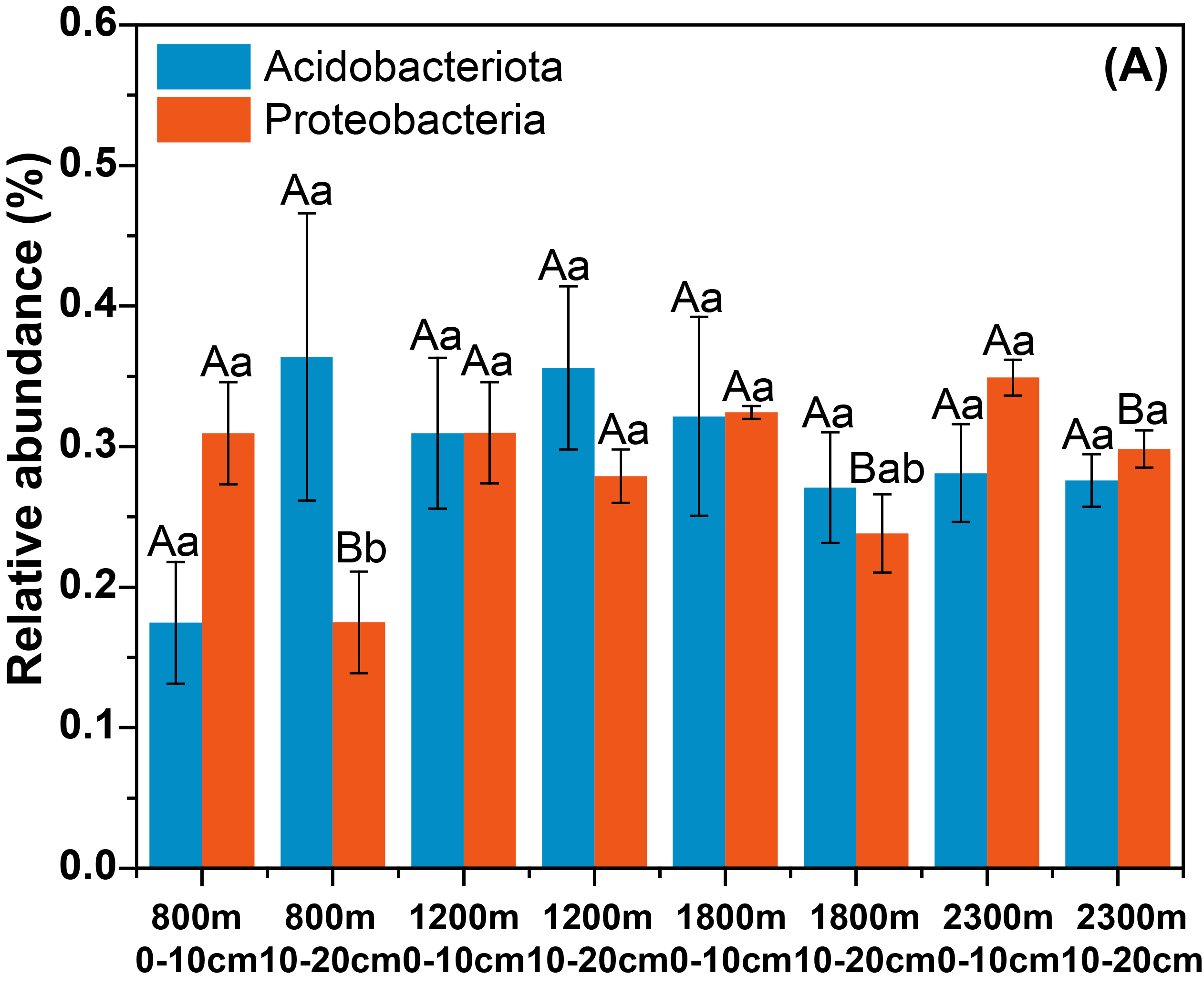

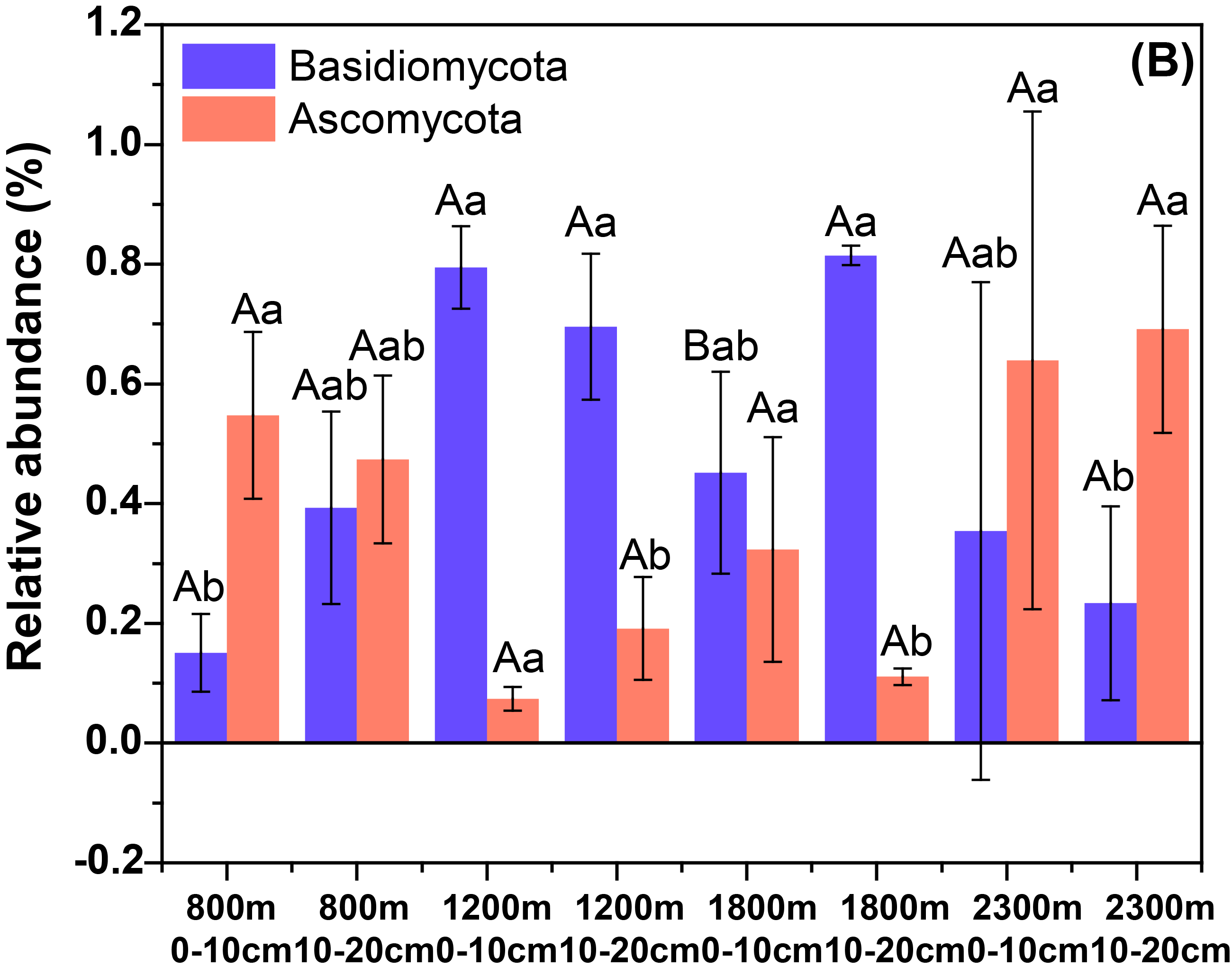


**Supplementary Figure 3.** Relative abundances of dominant phylum of bacteria (A) and fungi (B). Data are presented as means ± standard error (n = 3). Capital letters mean a statistical significance (p < 0.05) among two soil depths within the same altitude, and small letters mean a statistical significance (p < 0.05) along the different altitude within one soil depth.
